# MicroRNA expression profiles of drug-resistance breast cancer cells and their exosomes

**DOI:** 10.18632/oncotarget.7481

**Published:** 2016-02-18

**Authors:** Shanliang Zhong, Xiu Chen, Dandan Wang, Xiaohui Zhang, Hongyu Shen, Sujin Yang, Mengmeng Lv, Jinhai Tang, Jianhua Zhao

**Affiliations:** ^1^ Center of Clinical Laboratory Science, Jiangsu Cancer Hospital Affiliated to Nanjing Medical University, Nanjing 210009, China; ^2^ Department of General Surgery, Jiangsu Cancer Hospital Affiliated to Nanjing Medical University, Nanjing 210009, China

**Keywords:** microvesicles, breast, cancer, chemoresistance, microRNA

## Abstract

Exosomes have been shown to transmit drug resistance through delivering miRNAs. We aimed to explore their roles in breast cancer. Three resistant sublines were established by exposing parental MDA-MB-231 cell line to docetaxel, epirubicin and vinorelbine, respectively. Preneoadjuvant chemotherapy biopsies and paired surgically-resected specimens embedded in paraffin from 23 breast cancer patients were collected. MiRNA expression profiles of the cell lines and their exosomes were evaluated using microarray. The result showed that most miRNAs in exosomes had a lower expression level than that in cells, however, some miRNAs expressed higher in exosomes than in cells, suggesting a number of miRNAs is concentrated in exosomes. Among the dysregulated miRNAs, 22 miRNAs were consistently up-regulated in exosomes and their cells of origin. We further found that 12 of the 22 miRNAs were significantly up-regulated after preneoadjuvant chemotherapy. Further study of the role of these 12 miRNAs in acquisition of drug resistance is needed to clarify their contribution to chemoresistance.

## INTRODUCTION

Breast cancer is one of the most commonly diagnosed cancer and the leading cause of cancer death among females worldwide. Breast cancer alone accounts for 25% of all cancer cases and 15% of all cancer deaths among females [1]. Chemotherapy is commonly used in the treatment of breast cancers [2]. Nevertheless, many patients resistant to chemotherapy prior to drug treatment or develop resistance following treatment [3]. Despite much effort has been made, the underlying mechanisms of acquiring drug resistance are still poorly understood.

Recently, exosomes attract more attention as a novel mode of intercellular communication. Exosomes are small, lipid bilayer membrane vesicles 50 to 100 nm in diameter [4, 5]. Accumulating evidence indicates that exosomes play important roles in cancer, including tumorigenesis, growth, progression, metastasis, and drug resistance [4, 6, 7]. Exosomes can shuttle bioactive molecules including proteins and genetic cargo, especially low molecular weight RNA named microRNA (miRNA, miR) from one cell to another, leading to the exchange of genetic information and reprogramming of the recipient cells [6]. Our previously study suggested that drug-resistant breast cancer cells may spread resistance capacity to sensitive ones by releasing exosomes and that such effects could be partly attributed to the intercellular transfer of specific miRNAs [5, 8–10].

In present study, we established three drug resistance sublines from parental MDA-MB-231 cell line to explore miRNA expression profiles of drug-resistance breast cancer cells and their exosomes and their association with drug resistance. Preneoadjuvant chemotherapy biopsies and paired surgically-resected specimens from 23 breast cancer patients were collected and detected for the expression of 22 specific selected miRNAs to investigate whether these miRNAs are associated with drug resistance *in vivo*.

## RESULTS

### Sensitivity of MDA-MB-231 cells to anticancer drugs

To determine chemosensitivity of MDA-MB-231 cell lines to Doc, Epi and Nvb, the cell lines were treated with the drugs at different concentrations for 48h, cell viability was examined by MTT assay and 50% inhibition concentration (IC50) was calculated. The results showed that the resistant sublines were less sensitive than MDA-MB-231/S to the drugs used to select them and also cross-resistant to the other two drugs (Table 1).

**Table 1:** Antiproliferative effects of anticancer drugs on sensitive and resistant MDA-MB-231 cell lines

Cell lines	IC_50_±SEM		
	Docetaxel	Epirubicin	Vinorelbine
MDA-MB-231/S	2.75±0.26 μM	0.16±0.03 μM	7.71±3.91 nM
MDA-MB-231/Doc	22.89±4.71 μM	1.24±0.35 μM	0.54±0.08 μM
MDA-MB-231/Epi	6.92±1.90 μM	10.39±2.67 μM	0.18±0.03 μM
MDA-MB-231/Nvb	5.79±2.13 μM	0.89±0.12 μM	5.28±2.57 μM

IC50, 50% inhibition concentration; SEM, standard error of the mean.

### Exosomes characterization

**Figure 1:**
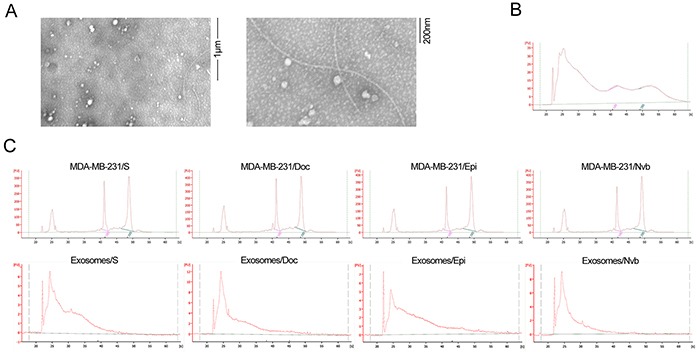
Electron microscopy of exosomes and RNA analyzed using a Bioanalyzer **A.** Representative micrograph of transmission electron microscopy of exosomes. **B.** Tissue RNA analyzed using a Bioanalyzer. **C.** Exosomal RNA and cell RNA analyzed using a Bioanalyzer. The results show that exosomes are enriched in small RNAs and contain no 18S and 28S ribosomal RNAs.

By transmission electron microscopy, exosomes appear with characteristic doughnut morphology, and their diameter ranges between 50 and 100 nm (Figure 1A). A bioanalyzer profile on total RNA from exosomes and their cells of origin revealed that RNAs isolated from exosomes were small-sized RNAs and lacked bands corresponding to cellular 18S and 28S ribosomal RNAs (Figure 1C).

### Expression profile of miRNAs

The miRNA expression profiles of the MDA-MB-231 cell lines and their exosomes were evaluated using Affymetrix GeneChip miRNA 4.0 Array. Supplementary Table S1 and Supplementary Table S2 present expression level of miRNAs in the four cell lines and their exosomes, respectively. Compared with MDA-MB-231/S cell line, there were respectively 121, 141 and 123 differentially expressed miRNAs (at least 2.0-fold changes) in MDA-MB-231/Doc, MDA-MB-231/Epi, and MDA-MB-231/Nvb cell lines. Regarding exosomes, 351, 216 and 112 differentially expressed miRNAs were found, respectively, in Exosomes/Doc, Exosomes/Epi, and Exosomes/Nvb when compared with Exosomes/S. Hierarchical cluster analysis revealed that the cell lines and their exosomes were characterized by significant changes in miRNA expression (Supplementary Figure S1).

The scatter-plots of the microarray signal values of between cell lines and between exosomes are shown in Supplementary Figure S2. The scatter plot that showed a correlation between the three resistant sublines and MDA-MB-231/S and between exosomes isolated from resistant sublines and Exosomes/S, although there were a lot of differentially expressed miRNAs. However, the scatter plot comparing the expression level of miRNAs between cells and exosomes showed that most miRNAs in exosomes had a lower expression level than that in cells, nevertheless, some miRNAs expressed higher in exosomes than in cells, suggesting that a number of miRNAs is concentrated in exosomes (Supplementary Figure S3).

**Figure 2:**
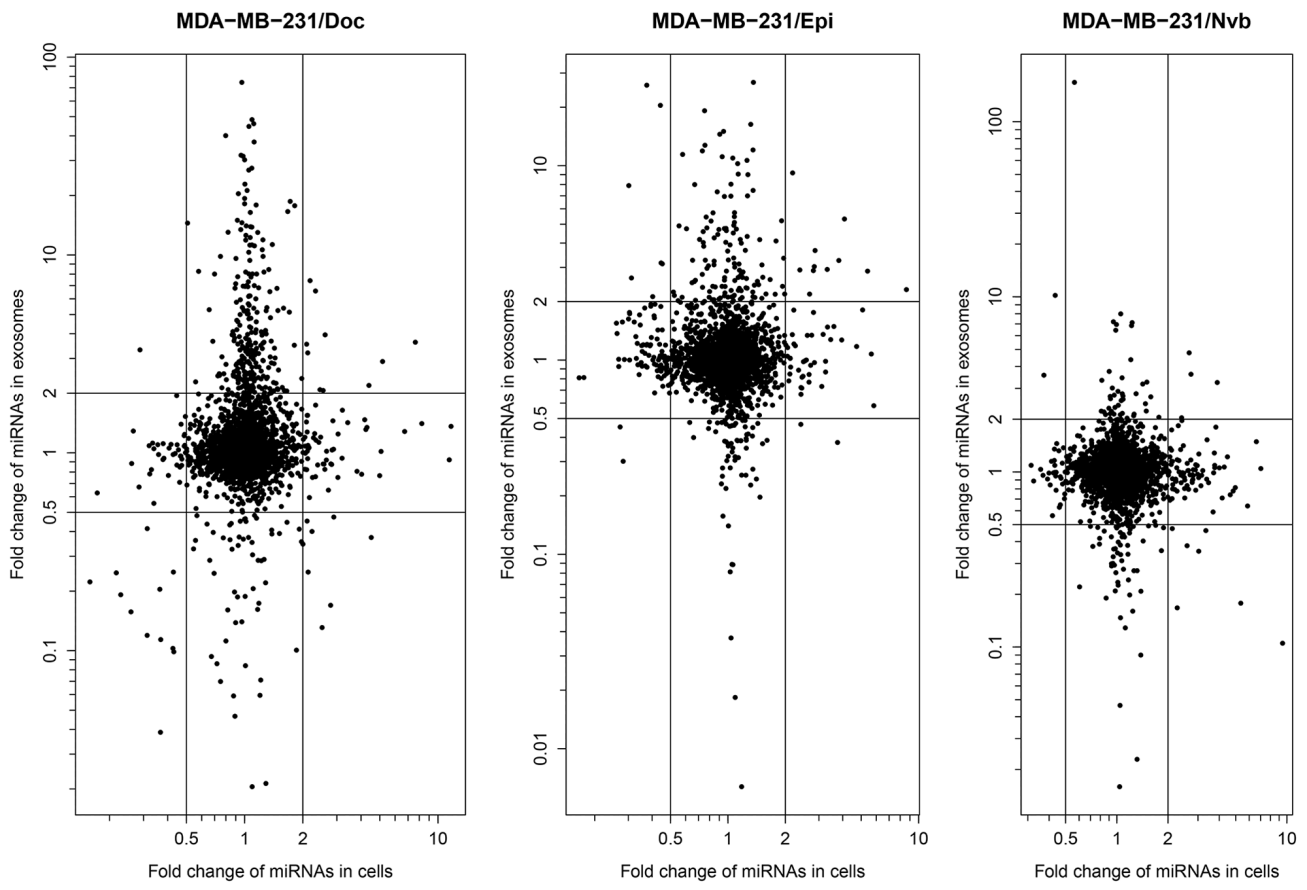
Fold change of miRNA expression level in the three resistance sublines relative to MDA-MB-231/S cell line vs. that in exosomes

Figure 2 presents fold change of miRNA expression level in the three resistance sublines relative to MDA-MB-231/S cell line vs. that in exosomes. From the Figure 2, we can easily found that some of the miRNAs exhibited similar or consistent expression changes in resistance cell lines and their exosomes, and the others either were dysregulated only in one of them, or showed changes in opposite directions. Among the dysregulated miRNAs, there were 10 consistently up-regulated miRNAs in MDA-MB-231/Doc cells and their exosomes (hsa-miR-1246, hsa-miR-1268a, hsa-miR-149-3p, hsa-miR-423-5p, hsa-miR-4298, hsa-miR-4438, hsa-miR-4644, hsa-miR-671-5p, hsa-miR-7107-5p and hsa-miR-7847-3p), 11 miRNAs in MDA-MB-231/Epi cells and their exosomes (hsa-miR-138-5p, hsa-miR-139-5p, hsa-miR-197-3p, hsa-miR-210-3p, hsa-miR-3178, hsa-miR-423-5p, hsa-miR-4258, hsa-miR-4443, hsa-miR-574-3p, hsa-miR-6780b-3p and hsa-miR-744-5p), and 4 miRNAs in MDA-MB-231/Nvb cells and their exosomes (hsa-miR-138-5p, hsa-miR-140-3p, hsa-miR-210-3p and hsa-miR-3613-5p). The following analyses will focus on these 22 miRNAs.

### RT-qPCR validation of microarray results

We used RT-qPCR to confirm the expression of the 22 consistently up-regulated miRNAs mentioned above. We failed to detect an expression of hsa-miR-671-5p in both cells and their exosomes. The RT-qPCR results of the rest 21 miRNAs showed good consistency with microarray results.

### MiRNA expression in patient samples

**Figure 3:**
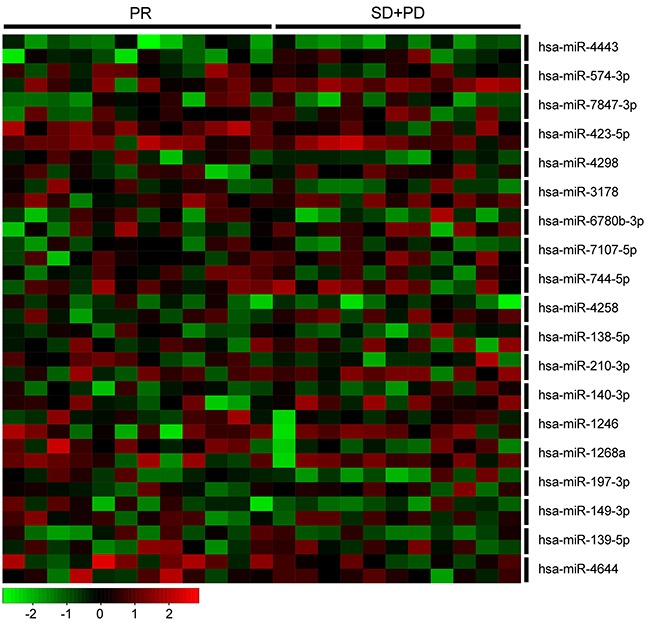
Heatmap of 19 miRNAs in 23 paired patient samples Every two horizontal rows represent a miRNA, and the first row represent its expression in preneoadjuvant chemotherapy biopsies and the second row represent its expression in paired surgically-resected specimens. Each vertical column corresponds to a sample. PR, partial response; SD, stable disease; and PD, progressive disease.

The characteristics of breast cancer patients (n=23), stratified by tumor response to neoadjuvant chemotherapy, are presented in Table 2. There were 12 patients of partial response (PR), 5 patients with stable disease (SD), and 6 patients with progressive disease (PD). We detected the expression of the 22 miRNAs in the 23 paired specimens from these patients to explore whether these miRNAs are associated with drug resistance *in vivo*. Because about a half of the specimens were failed to detect an expression of hsa-miR-3613-5p and hsa-miR-4438, and only 8 specimens were detected an expression of hsa-miR-671-5p, we removed the 3 miRNAs from present statistical analysis (Supplementary Table S3). Our results showed that the expression level of the rest 19 miRNAs was up-regulated after neoadjuvant chemotherapy, despite 7 miRNAs did not reach a statistical significance (Table 3, Figure 3). When we compared miRNA expression level in SD/PD group with that of in PR group, only hsa-miR-574-3p in surgically-resected specimens had significantly up-regulated expression (*P*=0.027).

**Table 2:** Characteristics of breast cancer patients

Characteristics	Tumor Response		
	PR (n=12)	SD (n=5)	PD (n=6)
Age, years			
≤55	4	2	2
>55	8	3	4
Treatment			
Docetaxel	9	4	6
Epirubicin	4	2	2
Pemetrexed disodium	6	3	3
Cytoxan	4	2	4
Stage at diagnosis			
I	3	3	0
II	5	2	1
III	4	0	5
Estrogen receptor			
Positive	6	4	2
Negative	6	1	4
Progesterone receptor			
Positive	4	4	2
Negative	8	1	4

PR, partial response; SD, stable disease; PD, progressive disease.

**Table 3:** Expression level of miRNAs and the results of Wilcoxon matched-pairs signed-rank test

miRNAs	Expression Level miRNAs (Median)^1^		*P*^2^
	Preneoadjuvant chemotherapy biopsies	Surgically-resected specimens	
hsa-miR-4443	0.00312	0.00521	0.001
hsa-miR-574-3p	0.02134	0.03768	0.005
hsa-miR-7847-3p	0.06493	0.11188	0.005
hsa-miR-423-5p	0.00187	0.00293	0.017
hsa-miR-4298	0.00600	0.01332	0.017
hsa-miR-3178	0.00136	0.00231	0.017
hsa-miR-6780b-3p	0.00018	0.00046	0.017
hsa-miR-7107-5p	0.02179	0.05366	0.017
hsa-miR-744-5p	0.00222	0.00376	0.017
hsa-miR-4258	0.00238	0.01024	0.047
hsa-miR-138-5p	0.00021	0.00069	0.047
hsa-miR-210-3p	0.00139	0.00302	0.047
hsa-miR-140-3p	0.00130	0.00227	0.067
hsa-miR-1246	0.15125	0.21464	0.105
hsa-miR-1268a	0.19547	0.27168	0.105
hsa-miR-197-3p	0.00115	0.00279	0.105
hsa-miR-149-3p	0.00867	0.02272	0.105
hsa-miR-139-5p	0.00012	0.00025	0.202
hsa-miR-4644	0.00016	0.00021	0.798

1 The expression level relative to U6.

2 *P* Value for one-tailed Wilcoxon matched-pairs signed-rank test for expression differences of miRNAs between preneoadjuvant chemotherapy biopsies and paired surgically-resected specimens.

### Analysis of involved pathways

**Figure 4:**
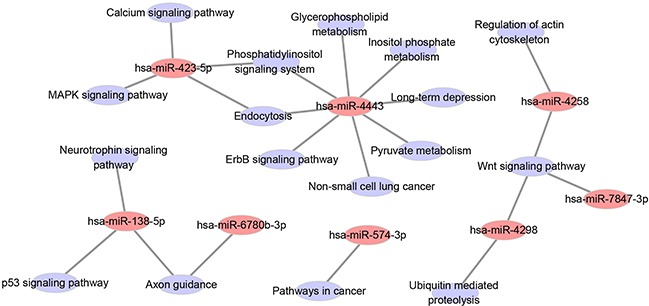
Network among the eight statistical significant miRNAs in patient samples and KEGG pathways of their predicted target genes

To identify which pathways might be involved in resistance formation, the target genes of the 12 miRNAs listed in Table 3 with a *P*-value <0.05 were compared with the whole reference gene background. Then, Cytoscape software V3.1.1 was used to decipher the KEGG pathway and understand their biological functions. The predicted target genes of the 8 miRNAs among the 12 miRNAs were enriched in 17 pathways with *P*-value less than 0.05 (Figure 4, Supplementary Table S4).

## DISCUSSION

MiRNA expression profile is a useful tool to acquire potential key miRNAs implicating in various aspects of cancer or other diseases. In previous study, we have screened different expressed miRNAs between parental MCF-7 cell line and drug resistant sublines using microarray and found miR-222 and miR-29a contributed to drug resistance of MCF-7 cells [13]. As our results showed that drug resistant breast cancer cells can deliver miRNAs to sensitive ones by releasing exosomes [5, 8, 10], exosomes carrying up-regulated miR-222 or/and miR-29a may be a transfer mechanism of resistance capacity to sensitive cells. Our following study has confirmed that the delivery of miR-222 via exosomes is a potential mechanism of transmitting drug resistance [9]. Besides miRNAs, exosomes also carry mRNA, enzymes or proteins, which might be another mechanism of spreading resistance capacity [19]. We found that delivering P-gp via exosomes could transmit drug resistance [12]. Taken together, drug resistant cells could spread chemoresistance to sensitive ones by exosomal miRNAs or proteins. In present study, MDA-MB-231 cell lines were used to explore the roles of exosomes and miRNAs in acquiring resistance to Doc, Epi, and Nvb.

We analyzed miRNA expression profiles of exosomes and their cells of origin. The results showed that most miRNAs in exosomes had a lower expression level than that in cells, however, some miRNAs expressed higher in exosomes than in cells, suggesting that a number of miRNAs is concentrated in exosomes. We hypothesis that miRNAs up-regulated in both resistance cell lines and their exosomes may play an important role in acquisition of resistance to chemotherapeutic agents. We obtained 22 consistently up-regulated miRNAs in exosomes and their cells of origin.

We further explored the 22 up-regulated miRNAs *in vivo*. Our results showed that 12 of the 22 miRNAs were significantly up-regulated after preneoadjuvant chemotherapy. However, only hsa-miR-574-3p was statistically up-regulated in SD/PD group compared to PR group. There are several potential reasons why the rest miRNAs did not reach a statistical significance. First, these included patients had different baseline expression levels of these miRNAs. Second, the patients vary in age, breast cancer stage, and chemotherapy regimen. Third, the effect of miRNAs *in vivo* may differ from *in vitro*. Fourth, the sample size is too small to detect statistical significance.

We employed pathway mapping tool to identify predominant pathways of the gene targets of the 12 miRNAs mentioned above. The result showed that the predicted target genes of 8 miRNAs were enriched in 17 pathways. Among the 8 miRNAs, hsa-miR-4443 interfering with maximum pathways may play more roles than the other miRNAs. Among the 17 pathways, p53 [20], Wnt [21], MAPK [22], and ErbB [23] signaling pathways have been confirmed to be involved in drug resistance. Down-regulation of the target genes following the four pathways in Supplementary Table S4 might be the potential mechanism of acquiring drug-resistance, which was worth further exploration by experimental studies. Whether or not other pathways and the following targets (Supplementary Table S4) also implicated in drug-resistance needs further investigation in the future.

In conclusion, we identified 12 miRNAs that may contribute to drug resistance of breast cancer. Drug-resistant breast cancer cells may spread resistance capacity to sensitive ones by releasing exosomes containing these miRNAs. Further studies uncovering the mechanisms of these miRNAs contributing to chemoresistance would help prevent or reverse chemoresistance in patients who receive chemotherapy for metastatic breast cancer.

## MATERIALS AND METHODS

### Cell lines and development of resistant sublines

Human breast cancer cell line MDA-MB-231 was purchased from the Cell Bank of the Chinese Academy of Sciences (Shanghai, China). The identities of cell lines were confirmed by the Cell Bank using DNA profiling (short tandem repeat, STR). Docetaxel (Doc) resistant MDA-MB-231 breast cancer subline (MDA-MB-231/Doc) was developed from the parental MDA-MB-231 cell line (MDA-MB-231/S) by increasing Doc concentration from 0.2 nM to 13 nM in our laboratory in about twelve months, epirubicin (Epi) resistant subline (MDA-MB-231/Epi) was established by exposing to Epi from 10 nM to 800 nM, and vinorelbine (Nvb) resistant subline (MDA-MB-231/Nvb) by exposing to Nvb from 10 nM to 1.4 μM. All the cell lines were cultured in Dulbecco's modified Eagle's medium (DMEM) high glucose (HyClone) supplemented with 10% fetal bovine serum (FBS), 100 U/mL penicillin, and 100 μg/mL streptomycin at 37°C and 5% CO_2_ in a humidified atmosphere. The drug resistant derivative cell lines were cultured in drug free medium for two weeks before subsequent experiments to avoid the influence of the drug.

### Cell proliferation inhibition assay

Cell proliferation was evaluated by 3-(4,5-dimethylthiazol-2-yl)-2, 5-diphenyltetrazolium bromide (MTT; Sigma) assay as previously described [11]. Briefly, MDA-MB-231 cells were plated at a density of 8×10^4^ cells mL^−1^ and dispensed into 96-well culture plate with 100μL of DMEM medium per well. After 24h, 100μL serial concentrations of the drugs were added to cultured cells (quadruplicate wells per condition). The cells were then cultured for 48h prior to the addition of 20μL of a 5 mg/mL solution of MTT into each well. The incubation was continued for 4h before the media was removed. 150μL dimethyl sulfoxide (DMSO; AMRESCO, America) was added to each well and mixed to ensure cell lysis and to dissolve the formazan crystal. At last, the absorbance at 550 nm was measured using CliniBio128 (ASYS-Hitech, Austria). Three replications of each experiment were performed.

### Isolation and identification of exosomes

Exosomes were isolated from culture medium of MDA-MB-231/S, MDA-MB-231/Doc, MDA-MB-231/Epi, and MDA-MB-231/Nvb cultured in exosome-free FBS prepared by ultracentrifugation at 110,000g for 16 hours. The terms Exosomes/S, Exosomes/Doc, Exosomes/Epi, and Exosomes/Nvb in the following text refers to exosomes extracted from culture medium of corresponding MDA-MB-231 cells. Exosomes were isolated using repeated centrifugation, and ultracentrifugation. Briefly, the culture medium was centrifuged at 2,000g for 15 minutes to eliminate cells, and then centrifuged at 16,500g for 30 minutes to get rid of debris. Exosomes were pelleted by ultracentrifugation at 110,000g for 120 minutes. The final pellets, comprised of exosomes, were used immediately or resuspended in 1mL PBS and stored at −80°C.

The exosomes were confirmed under electron microscopy as our previous reports [5, 8, 12]. In short, 10μL exosomes were dripped onto parafilm and covered by a 300-mesh copper grid for 45 min. After washing three times with PBS, the copper mesh was fixed in 3% glutaraldehyde for 10 min, washed with double distilled water, and contrasted in 2% uranyl acetate. Images were captured by a JEM-1010 electron microscope (JEOL, Japan) at an accelerating voltage of 80 kV.

### Isolation and profiling of miRNA from exosomes and their cells of origin

Cells in logarithmic phase were collected when a confluence of 80-90% was reached, and then total RNA including miRNAs was extracted from the cells using MirVana miRNA isolation Kit (Ambion, USA). RNA recovery from the exosome samples was performed with the Total Exosome RNA and Protein Isolation Kit (Invitrogen, USA), following the manufacturer's protocol. RNA was quantified spectrophotometrically (Thermo Scientific, USA), and its quality was assessed by an Agilent 2100 Bioanalyzer (Agilent Technologies, USA).

Microarray hybridization and analysis were performed as previously described [11, 13], using Affymetrix GeneChip miRNA 4.0 Array, which contains 2578 human mature miRNA probe sets. The data output was received in Excel spreadsheets containing the normalized miRNA expression profiles. Differentially expressed miRNAs were filtered to exclude those changes less than 2.0-fold compared with MDA-MB-231/S or Exosomes/S.

### Real-time quantitative PCR (RT-qPCR)

Expression of miRNA was analyzed using MiR-X miRNA qRT-PCR SYBR Kit (638314; Clontech Laboratories, USA) on Roche LightCycler 480 II. U6 snRNA was used as endogenous control to normalize miRNA expression in cells and tissues. The primers for U6 were 5′- CTCGCTTCGGCAGCACA-3′ and 5′- AA CGCTTCACGAATTTGCGT-3′. MiR-16 was used as endogenous control to normalize miRNA expression in exosomes. The forward primers of miR-X corresponds to the miR-X sequence but with the substitution of uridine for thymidine. The Ct values for each gene were normalized to endogenous control, and the relative fold change values were calculated using the ΔΔCt method. Every run experiment was performed along with negative controls (nuclease-free water or the extracted RNA without RT as a template). PCR specificity was judged based on the presence of a single product at the end of the 40 cycles based on melting curve analyses showing a single peak at the product melting temperature, as well as based on examination of the products after 2% agarose gel electrophoresis. All the samples were analyzed in duplicates for each specific gene.

### Patients and samples

We retrospectively identified 23 patients who underwent neoadjuvant chemotherapy, followed by surgery for primary breast cancer at Nanjing Drum Tower Hospital between January 2010 and February 2015. The preneoadjuvant chemotherapy biopsies and paired surgically-resected specimens of these patients, routinely fixed in 10% formalin and embedded in paraffin, were obtained from the Department of Pathology. Medical records were reviewed to obtain information about patient, tumor, and treatment characteristics. Tumor response to neoadjuvant chemotherapy was evaluated based on the Response Evaluation Criteria in Solid Tumors (RECIST) guidelines [14]. The study protocol was approved by the ethics committee of Nanjing Drum Tower Hospital, and informed consent was obtained from all patients.

### Isolation miRNA from formalin-fixed paraffin-embedded tissues

Ten serial 10-μm-thick sections were prepared from each paraffin-embedded tissue block. One paraffin section was stained using hematoxylin and eosin (H&E) to mark out tumorous breast tissues by a skilled pathologist. Total RNA was extracted from tumorous breast tissues of three paraffin sections using Recover All Total Nucleic Acid Isolation Kit (Ambion, Carlsbad, CA, USA) according to the manufacturer's instructions. RNA was quantified and RT-qPCR was performed using the method described above. Three replications of each experiment were performed.

### Prediction of target genes

The target genes of the specific selected dysregulated miRNAs were predicted by in silico analysis using TargetScan [15] (Release 7.0, http://www.targetscan.org) and miRDB [16] (Version 5.0, http://www.mirdb.org). Given that the prediction programs often suffer from high false positive rates, only the target genes predicted by both programs were considered further.

### KEGG pathway analysis of target genes

KEGG (Kyoto Encyclopedia of Genes and Genomes) pathway enrichment analysis was performed to compare the specific miRNAs targets with the whole reference gene background using Database for Annotation, Visualization and Integrated Discovery [17] (DAVID, Version 6.7, http://david.abcc.ncifcrf.gov). The count number lager than 2 and *P*-value less than 0.05 were chosen as cut-off criterion. Cytoscape 3.1.1 was utilized to construct the possible functional network of the selected miRNAs [18].

### Data analysis

Statistical significance of miRNA expression between preneoadjuvant chemotherapy biopsies and paired surgically-resected specimens was assessed using Wilcoxon matched pairs signed rank test. Wilcoxon-Mann-Whitney rank sum test was used to evaluate expression differences of miRNAs between different groups of cell lines or patients. Statistical significance was assumed when *P* < 0.05. Statistical analysis was performed using R software, version 3.1.1.

## SUPPLEMENTARY FIGURES AND TABLES








