# Supplementary material for: MicroRNA expression profiles of drug-resistance breast cancer cells and their exosomes
Source: Oncotarget. 2016 Feb 18;7(15):19601–9. doi: 10.18632/oncotarget.7481 (PMC4991404; doi:10.18632/oncotarget.7481)
Supplement: Supplementary file 1 [file oncotarget-07-19601-s001.pdf]

SUPPLEMENTARY FIGURES AND TABLES

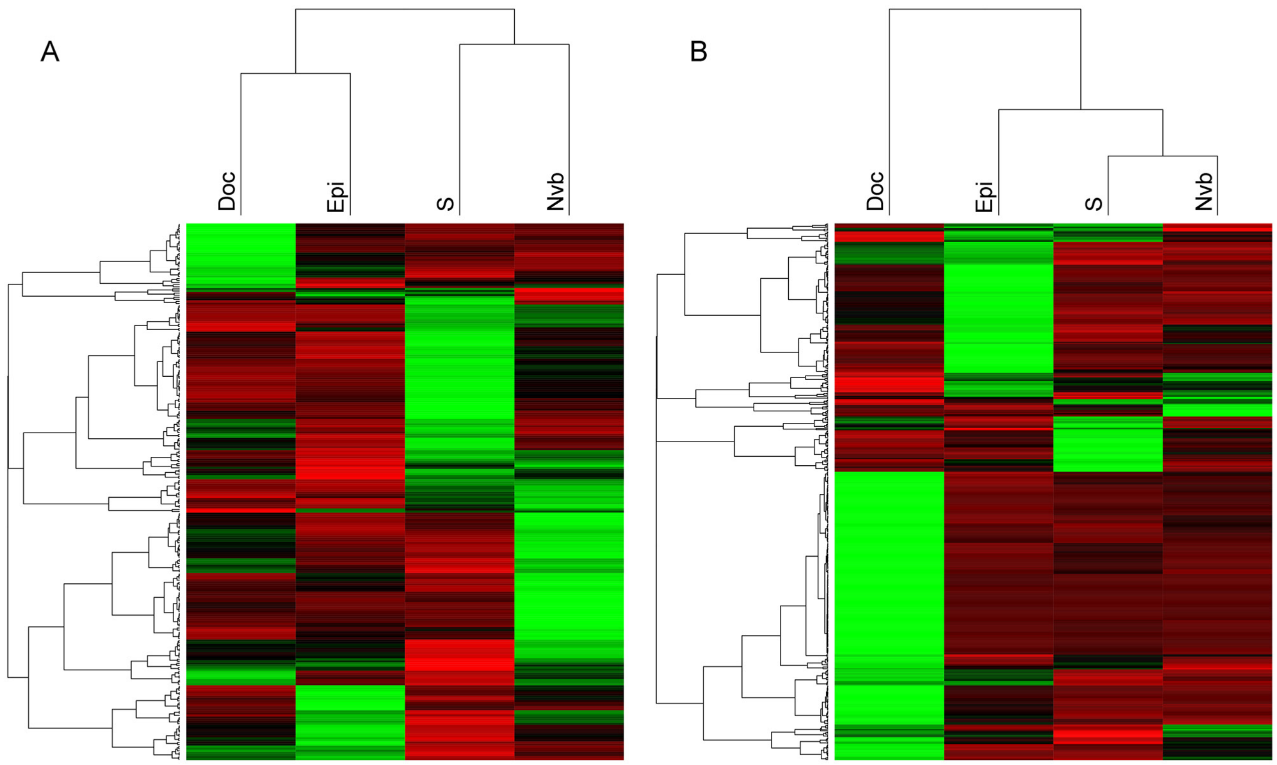

Supplementary Figure S1: Hierarchical cluster analysis of differentially expressed miRNAs in **A.** MDA-MB-231 cell lines and **B.** their exosomes. Each horizontal row represents a miRNA, and each vertical column corresponds to a sample.

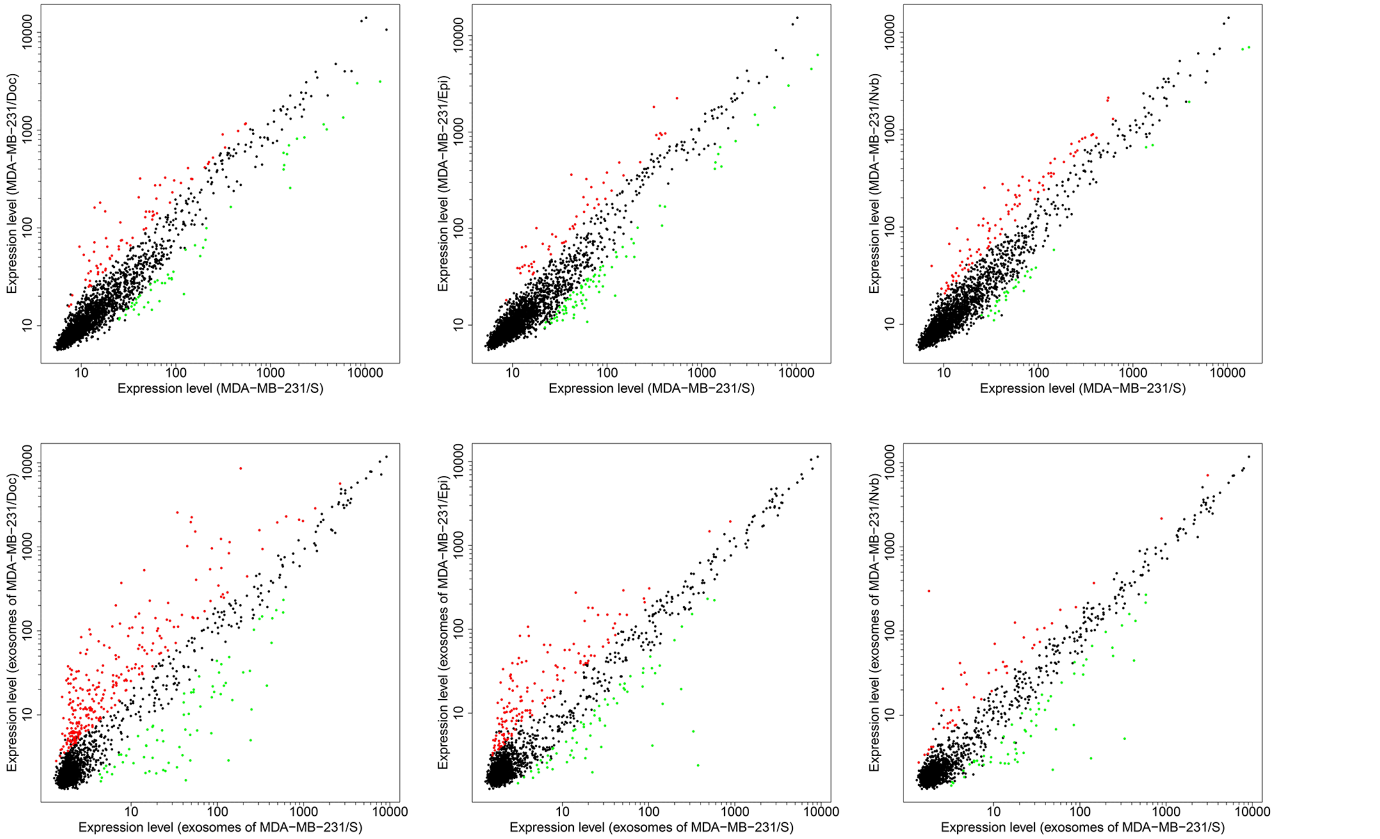

Supplementary Figure S2: The scatter-plots of the microarray signal values of between cell lines and between exosomes. Red dots: up-regulated miRNAs compared to MDA-MB-231/S or Exosomes/S (fold-change>2); Green dots: down-regulated miRNAs compared to MDA-MB-231/S or Exosomes/S (fold-change>2); Black dots: equally expressed miRNAs (fold-change≤2).

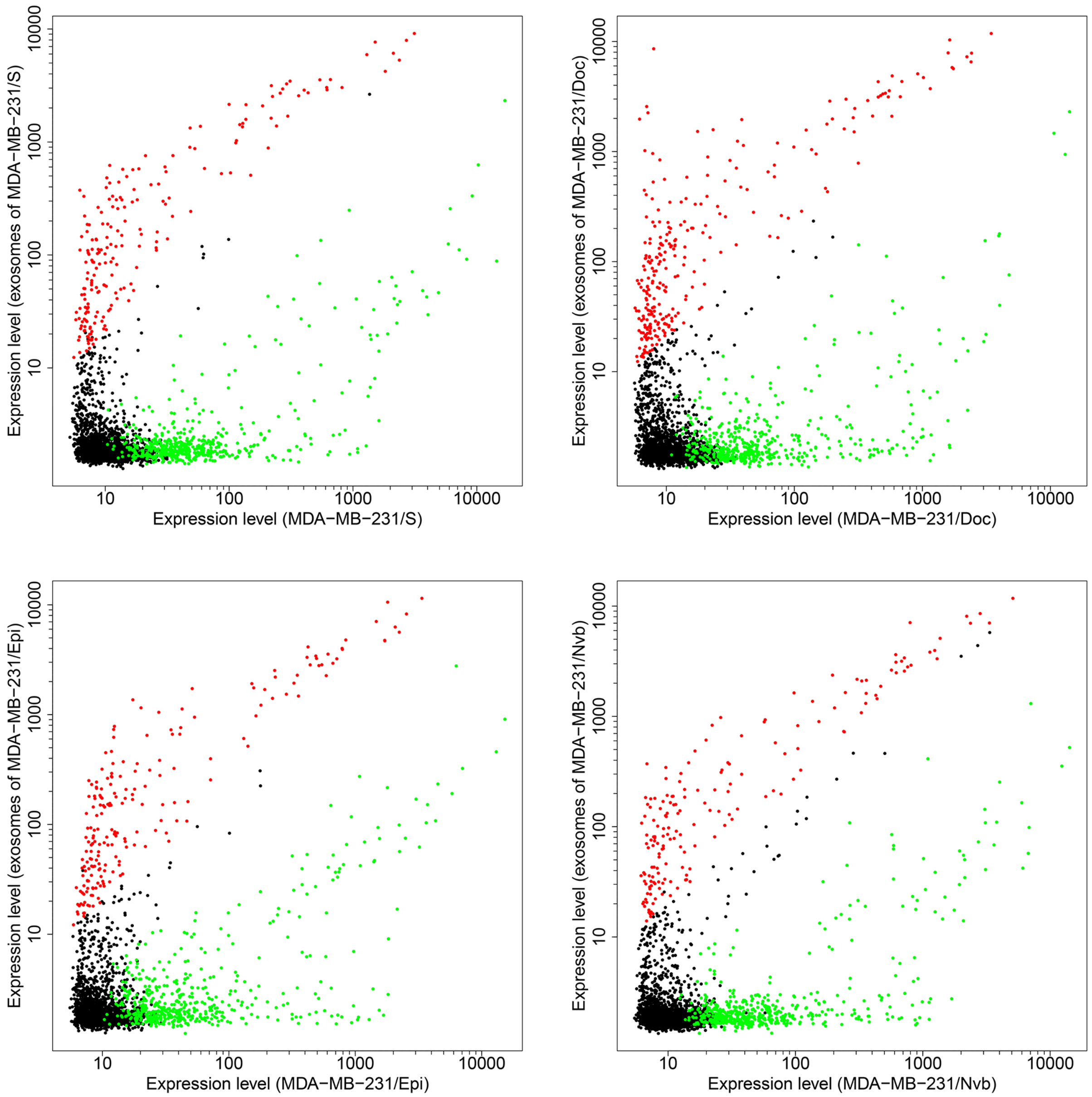

Supplementary Figure S3: The scatter-plots of the microarray signal values of between cell lines and their exosomes. Red dots: up-regulated miRNAs in exosomes compared with their cell of origin (fold-change>2); Green dots: down-regulated miRNAs in exosomes compared with their cell of origin (fold-change>2); Black dots: equally expressed miRNAs (fold-change≤2).

Supplementary Table S1: Expression level of miRNAs in MDA-MB-231 cell lines from Affymetrix GeneChip miRNA 4.0 Array

See Supplementary File 1

Supplementary Table S2: Expression level of miRNAs in exosomes of MDA-MB-231 cell lines from Affymetrix GeneChip miRNA 4.0 Array

See Supplementary File 2

Supplementary Table S3: ΔCt values relative to U6 of 22 miRNAs in 23 patients

See Supplementary File 3

Supplementary Table S4: KEGG pathway enrichment analysis with DAVID tool

miRNA	Term	Gene Count (%)	*P*-Value	Genes
hsa-miR-138-5p	Axon guidance	11 (3.16)	0.000	EPHA4, PTK2, KRAS, EFNB3, ROCK2, EPHA8, UNC5A, PLXNB2, SEMA4C, UNC5D, NTN1
	Neurotrophin signaling pathway	7 (2.01)	0.023	KRAS, RPS6KA1, SH2B3, SORT1, CAMK2A, TP73, PTPN11
	p53 signaling pathway	5 (1.44)	0.033	CCNE1, SHISA5, CDK6, TP73, SESN3
hsa-miR-423-5p	Calcium signaling pathway	8 (0.30)	0.025	EGFR, PTK2B, GRIN1, ADRA1B, PTGFR, NTSR1, CACNA1S, CALM1
	Endocytosis	8 (0.30)	0.031	EGFR, ARFGAP2, FGFR3, VPS37A, RAB11B, PIP5K1C, IQSEC3, EHD3
	MAPK signaling pathway	11 (0.42)	0.012	EGFR, FGFR3, GNA12, MKNK2, CACNB1, PLA2G6, NFATC4, DUSP8, CACNA2D2, CACNA1S, NGF
	Phosphatidylinositol signaling system	5 (0.19)	0.033	DGKQ, PIP5K1C, PI4KB, CALM1, PIK3R2
hsa-miR-4258	Regulation of actin cytoskeleton	7 (3.29)	0.043	APC2, LIMK1, RAC3, FGF17, NCKAP1L, PIK3R5, CSK
	Wnt signaling pathway	6 (2.82)	0.034	CSNK2A1, WNT5B, APC2, RAC3, WNT8A, PRKX
hsa-miR-4298	Ubiquitin mediated proteolysis	5 (3.62)	0.030	UBE2O, CUL5, HUWE1, UBA6, SKP1
	Wnt signaling pathway	5 (3.62)	0.040	PPARD, NKD1, CTBP2, CXXC4, SKP1
hsa-miR-4443	Endocytosis	19 (2.03)	0.003	ARFGAP1, FGFR3, ERBB4, LDLR, PSD3, PIP5K1C, ADRBK1, PIP5K1A, RAB11FIP4, ACVR1B, RAB11FIP5, RAB11FIP3, PSD, TFRC, IQSEC3, CLTCL1, AGAP2, EHD4, EPN2
	ErbB signaling pathway	10 (1.07)	0.024	PRKCA, PAK6, PAK7, EIF4EBP1, ERBB4, PLCG1, GRB2, PIK3CD, TGFA, ABL2
	Glycerophospholipid metabolism	9 (0.96)	0.016	DGKA, DGKQ, DGKG, LYPLA2, ETNK2, AGPAT4, PLA2G2D, PLA2G4E, PLA2G2F

(*Continued*)

miRNA	Term	Gene Count (%)	*P*-Value	Genes
	Inositol phosphate metabolism	8 (0.85)	0.014	PLCG1, INPP5K, PIK3C2B, PIK3CD, SYNJ1, PIP5K1C, PIP5K1A, ITPK1
	Long-term depression	8 (0.85)	0.048	PRKCA, PPP2R1B, NOS1, GUCY1A2, PLA2G2D, PLA2G4E, ITPR2, PLA2G2F
	Non-small cell lung cancer	8 (0.85)	0.014	PRKCA, RASSF5, PLCG1, GRB2, RXRB, PIK3CD, TGFA, RARB
	Phosphatidylinositol signaling system	13 (1.39)	0.000	PRKCA, DGKQ, PIK3C2B, SYNJ1, PIK3CD, PIP5K1C, PIP5K1A, ITPR2, DGKA, INPP5K, PLCG1, DGKG, ITPK1
	Pyruvate metabolism	6 (0.64)	0.042	ALDH7A1, LDHAL6A, ACACA, PDHA1, ACSS2, PCK1
hsa-miR-574-3p	Pathways in cancer	3 (21.43)	0.004	CUL2, ACVR1B, RXRA
hsa-miR-6780b-3p	Axon guidance	4 (3.77)	0.024	SEMA5A, NRAS, NRP1, NFAT5
hsa-miR-7847-3p	Wnt signaling pathway	12 (0.34)	0.000	CTNNBIP1, WNT5B, PRICKLE1, NLK, PRICKLE2, NFAT5, AXIN2, NFATC2, TBL1X, FOSL1, NFATC3, WNT2B
